# Epithelial–Mesenchymal Transition Suppression by ML210 Enhances Gemcitabine Anti-Tumor Effects on PDAC Cells

**DOI:** 10.3390/biom15010070

**Published:** 2025-01-06

**Authors:** Keisuke Takemura, Kyohei Ikeda, Hayato Miyake, Yoshio Sogame, Hiroaki Yasuda, Nobuhiro Okada, Kazumi Iwata, Junichi Sakagami, Kanji Yamaguchi, Yoshito Itoh, Atsushi Umemura

**Affiliations:** 1 Department of Pharmacology, Kyoto Prefectural University of Medicine, 465 Kajii-cho, Kawaramachi-Hirokoji, Kamigyo-ku, Kyoto 602-8566, Japan; tak1016@koto.kpu-m.ac.jp (K.T.); kikeda@koto.kpu-m.ac.jp (K.I.); okadan@koto.kpu-m.ac.jp (N.O.); iwatak@koto.kpu-m.ac.jp (K.I.); 2 Department of Molecular Gastroenterology and Hepatology, Graduate School of Medical Science, Kyoto Prefectural University of Medicine, 465 Kajii-cho, Kawaramachi-Hirokoji, Kamigyo-ku, Kyoto 602-8566, Japan; mi3ya4ke@koto.kpu-m.ac.jp (H.M.); sogame@koto.kpu-m.ac.jp (Y.S.); hiyasuda@koto.kpu-m.ac.jp (H.Y.); junichi@koto.kpu-m.ac.jp (J.S.); ykanji@koto.kpu-m.ac.jp (K.Y.); yitoh@koto.kpu-m.ac.jp (Y.I.); 3 Saiseikai Shiga Hospital, 2-4-1 Ohashi, Ritto 520-3046, Shiga, Japan; 4 Fukuchiyama City Hospital, 231 Atsunaka-cho, Fukuchiyama 620-8505, Kyoto, Japan

**Keywords:** pancreatic ductal adenocarcinoma, neoadjuvant chemotherapy, epithelial–mesenchymal transition, ML210, gemcitabine

## Abstract

Pancreatic ductal adenocarcinoma (PDAC) is one of the deadliest cancers in the world. Neoadjuvant chemotherapy (NAC) has become a standard treatment for patients scheduled for surgical resection, but the high rate of postoperative recurrence is a critical problem. Optimization of NAC is desirable to reduce postoperative recurrence and achieve long-term survival. However, if a patient’s general condition deteriorates due to NAC toxicity, surgical outcomes may be compromised. Therefore, we aimed to identify drug(s) that can be used in combination with gemcitabine (GEM), a drug widely used for the treatment of PDAC, to inhibit distant metastatic recurrence, particularly after surgery. After several screening steps, ML210, a low molecular weight chemical, was found to suppress the epithelial–mesenchymal transition (EMT) in PDAC cells in combination with GEM. Specifically, low dose ML210 in combination with GEM was sufficient for cell migration without apparent toxicity or cell death. Mechanistically, ML210, which was developed as a glutathione peroxidase 4 (GPX4) inhibitor to induce lipid peroxidation, increased the oxidized lipid concentrations in PDAC cells. The oxidization of the cell membrane lipids may suppress EMT, including cell migration. Since EMT is a major malignant phenotype of PDAC, our findings may lead to the advancement of PDAC therapy, especially in the prevention of postoperative recurrence.

## 1. Introduction

Pancreatic ductal adenocarcinoma (PDAC) is the seventh leading cause of cancer death worldwide [1]. With an increasing incidence [2] and a low survival rate of 12% [3] that has remained largely stagnant for nearly 60 years [3], PDAC is projected to cause even greater numbers of global cancer deaths by 2025 [2,4]. Pancreatectomy is the only curative treatment for PDAC; however, nearly 90% of patients experience disease recurrence at a median of 7–9 months after surgery [5,6], and the 5-year overall survival (OS) rate is only 8–10% [5,6]. Although adjuvant multi-agent chemotherapies delay recurrence and are the standard of care in surgically resected PDAC, approximately 80% of patients receiving adjuvant therapy experience disease recurrence at around 14 months [7], and their 5-year OS rate is <30%, demonstrating the need for a more efficient treatment strategy [8]. Very recently, neoadjuvant chemotherapy (NAC, preoperative chemotherapy) has improved curative resection rates by approximately 20% in patients with resectable PDAC [9]. Currently, a combination of gemcitabine (GEM) and S–1 is widely used as a standard NAC regimen; however, better treatment regimens or drugs that are effective but have low enough toxicity to permit surgery and achieve longer survival without postoperative recurrence are strongly desired.

In addition to cancer cells, many types of cells also undergo an epithelial-to-mesenchymal transition (EMT) to adopt a migratory program [10]. For PDAC cells, EMT and migration are important targets [11]. Recurrence and metastasis are prognostic factors after NAC/pancreatectomy, and EMT and migration are the most important factors. The use of EMT to adopt a migratory program is a phenomenon in which epithelial cells acquire mesenchymal traits, and cancer cells that have undergone EMT become poorly differentiated/undifferentiated and acquire characteristics favorable for migration and metastasis, such as increased motility [11]. This is one of the major factors controlling the malignancy and disease progression of cancer, including metastasis and peritoneal dissemination [12]. It has also been reported that pancreatic cancer cells exhibiting the EMT phenotype are resistant to chemotherapy [13]. Therefore, therapeutic agents targeting EMT, used alone or in combination with anticancer agents currently used for PDAC, may lead to improved therapeutic efficacy and outcomes of NAC/pancreatectomy and chemotherapy in pancreatic cancer treatment and contribute to improved patient prognosis. Recently, Moffitt Ra et al. reported that pancreatic cancer was divided into basal-like and classical types based on tumor-specific gene expression, and the basal-like type was characterized by activated Kras and low GATA6 expression and was considered to have a poor prognosis [14]. Our study may contribute to improving the treatment outcomes of the basal-like type, which is known to have a poor prognosis.

Since both GEM and S–1 have little effect on EMT and EMT causes GEM resistance [15], pancreatic cancer treatment would benefit if drugs with inhibitory effects on EMT were used. Effective NAC should rely on the use of safe, low-cost drugs, including newly synthesized or naturally occurring compounds.

## 2. Experimental Procedures

### 2.1. Human PDAC Cells

PANC-1 cells and MiaPaCa-2 cells were maintained according to the ATCC’s instructions and incubated with ML210 with/without GEM at the indicated concentrations and time course. For analysis of cell viability, CellTiter-Glo Luminescent Cell Viability Assay (Promega, Tokyo, Japan), a homogeneous method to determine the number of viable cells in culture based on quantitation of the ATP present, which signals the presence of metabolically active cells, was used according to the manufacturer’s instructions. To assess drug-induced cytotoxicity, LDH release was measured in cell culture supernatants using the LDH Cytotoxicity Assay kit (Nacalai tesque, Kyoto, Japan) according to the manufacturer’s protocol. The absorbance at λ = 490 nm was measured using a microplate reader. Gemcitabine and ML210 were purchased from FUJIFILM Wako Pure Chemical Corporation, Osaka, Japan (#073-06631) and Selleck Biotechnology, Osaka, Japan (#S0788), respectively. Each drug was used at the indicated concentrations, and RNA or protein extraction was performed 48 h later.

### 2.2. Drug Screening and Real-Time Quantitative (q) PCR

Drug screening was performed by real-time quantitative (q) PCR. RNA was extracted from the PANC-1 cells by using a SuperPrep2 kit (TOYOBO, Osaka, Japan). Then, cDNA was synthesized and used as a template for real-time qPCR. We used two drug libraries, 184 flavonoid- and 101 covalent-compounds, for the first screening.

The drug libraries (Flavonoid Compound Library [L7700] and Covalent Inhibitor Library [L5800]) were purchased from Selleck Biotechnology (Osaka, Japan). The gene expression level of vimentin was used as the marker and two compounds, ML210 and CA-074 methyl ester, were selected because their vimentin expression was strongly downregulated.

Individual gene expression was quantified by the TB Green Premix Ex taq 2 (Takara Bio Inc, Shiga, Japan) and ABI7300 Real-Time PCR system (Applied Biosystems, Woburn, MA, USA), then normalized to a housekeeping control gene, GAPDH. Primer sequences are as follows: vimentin (Forward: TCCAAACTTTTCCTCCCTGAAC and Reverse: GGGTATCAACCAGAGGGAGTGA), N-cadherin (Forward: TGGGAATCCGACGAATGG and Reverse: GCAGATCGGACCGGATACTG), GATA6 (Forward: GTGCCCAGACCACTTGCTAT and Reverse: TGGAATTATTGCTATTACCAGAGC), and GAPDH (Forward: TGACAACTTTGGTATCGTGGAAGG and Reverse: AGGCAGGGATGATGTTCTGGAGAG). Target gene levels were presented as a ratio of levels in the treated versus corresponding control groups.

### 2.3. Migration Assay

A total of 2 × 10^5^ PANC-1 cells and MiaPaCa cells were spread on 6-well dish plates and incubated for 24 h. The cells were then treated with 3 mg/L GEM + 0/0.025/0.05/0.1/0.3μM ML210 for 24 h. The treated cells were trypsinized, suspended in FBS (−) medium, and seeded into the culture inserts with a 8.0 µm filter membrane (Corning, NewYork, NY, USA #353097). The culture inserts were placed on the bottom of chambers containing 750 μL of FBS (+) medium. After a 24 h incubation, the filter membranes were fixed with cold methanol and stained with crystal violet for 15 min. The cells on the upper surface of filter membranes were gently removed with a cotton swab, and the cells on the lower surface of the membranes were analyzed under a BZ-X800 microscope with the imaging system (Keyence Corporation, Osaka, Japan). All experiments were repeated three times.

### 2.4. Protein Immunoblotting

PANC-1 cells and MiaPaCa cells were homogenized in RIPA buffer and then equal amounts of cell homogenates were fractionated by SDS-PAGE and transferred onto a PVDF membrane according to our previous report [16]. The membrane was incubated with antibodies to tubulin (Sigma-Aldrich, St. Louis, MO, USA #T9026), vimentin (Santa Cruz Biotechnology, Dallas, TX, USA, #6260), GPX4 (Abcam, Cambridge, England, #125066), and cleaved caspase-3 (Cell Signaling Technology, Danvers, MA, USA, #9664).

### 2.5. Lipid-Peroxidation Assay

A total of 2 × 10^5^ PANC-1 cells were incubated with ML210 at the concentrations 0/0.1/0.25/0.5 µM for 24 h. Tert-butyl hydroperoxide (tBHP) was used as a positive control drug for inducing lipid peroxidation. After washing with PBS, 10 µM of BODIPY 581/591 C11 (Thermo Fisher Scientific, Waltham, MA, USA, D3861) was added. After the incubation for 30 min, treated cells were collected in FACS tubes then centrifuged at 300× *g* for 5 min. After resuspending with PBS-3% FBS and filtration with a 40 µm filter, the cells were analyzed with a BD FACS Celesta flow cytometer (Excitation 490, Fluorescence 520–540 FITC) (BD Life Sciences-Biosciences, Franklin Lakes, NJ, USA). All experiments were repeated five times.

### 2.6. Statistical Analysis

Results obtained in the experiments are presented as the means ± standard deviations. Comparisons were made by a two-tailed t test or Dunnett’s multiple comparison test to calculate whether observed differences were statistically significant. Results with *p* values less than 0.05 were considered significant.

## 3. Results

### 3.1. ML210 and CA-074 Methyl Ester Attenuate Vimentin and N-Cadherin Expression in PDAC Cells

For NAC of PDAC, low-toxicity compounds or drugs sufficiently potent at low concentrations are likely to be more appropriate than cytotoxic anticancer drugs. In addition, safe drugs should be suitable for combination therapy with GEM, which is one of the most widely used drugs for patients with PDAC that has a known profile [17]. Cancer cells with an EMT phenotype are poorly differentiated/undifferentiated and possess a high ability for migration/invasion and metastasis [11]. In addition, EMT is also a factor in anti-cancer drug resistance, including resistance to GEM [15]. Therefore, drugs exerting suppressive effects on EMT can be highly beneficial for patients with PDAC. In the present study, we screened two chemical libraries including 184 flavonoid compounds and 101 covalent compounds to find potential agents that could inhibit EMT.

**Figure 1:**
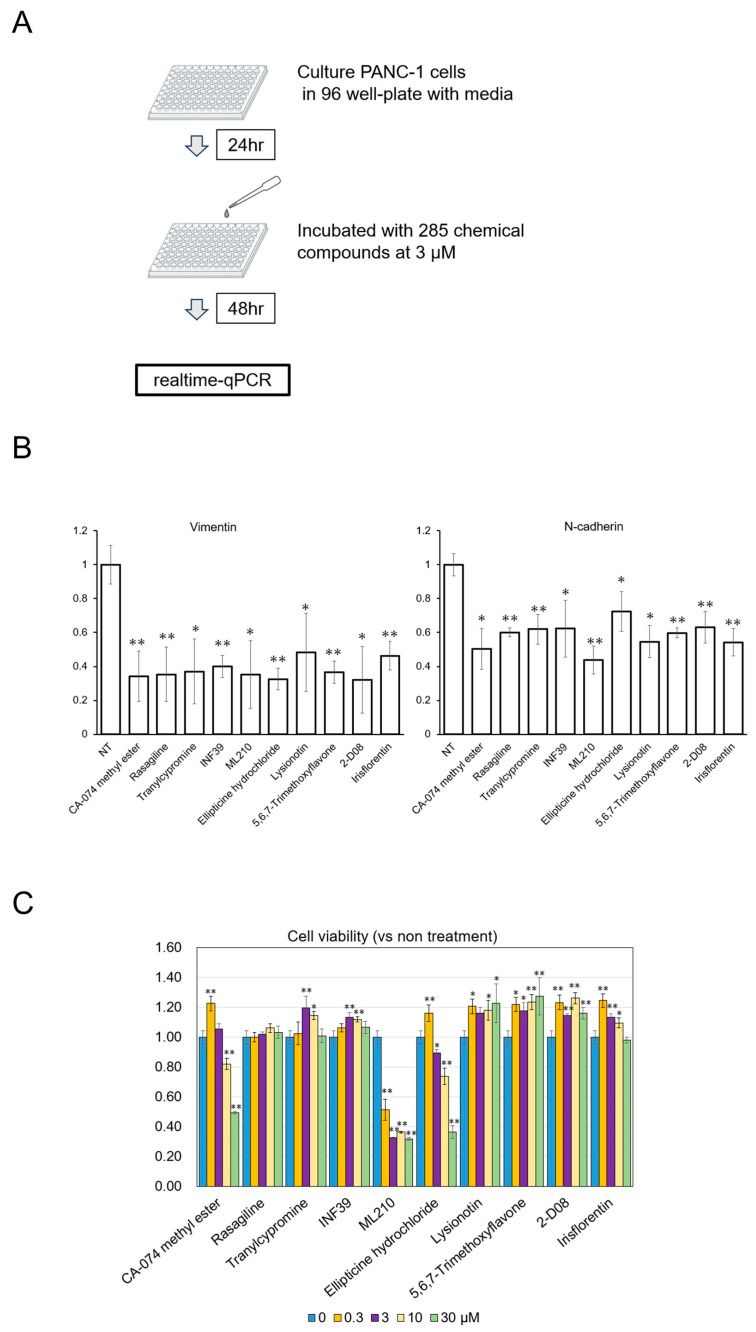
Drug screening. (**A**) Schematic explanation for the drug screening process. A total of 10,000 cells were seeded in a 96-well dish with 100 µL of culture medium, and we replaced the medium with each drug compound at concentrations of 3 µM after 24 h. The cells were harvested after a 48 h incubation. (**B**) Vimentin and N-cadherin gene expression analyzed by real-time qPCR. (**C**) Cell viability of PANC-1 cells. A total of 5000 cells/well (100 μL) were treated with each compound at the indicated concentrations (0.3, 3, 10, 30 μM), and their viabilities were assessed after a 48 h incubation (vs. non-treatment control). Data were obtained from three independent experiments. Mean ± SD data are displayed as fold changes relative to the non-treated group. * *p* < 0.05, ** *p* < 0.01 (vs. the non-treated group).

After human-derived PDAC cells (PANC-1 cells) were treated with 3 µM of each chemical for 48 h, expressions of the mRNA levels of mesenchymal markers of EMT were measured by real-time quantitative PCR (RT-qPCR, Figure 1A). Among the 285 total chemicals, 10 showed decreased expression levels of vimentin and N-cadherin, major mesenchymal markers, compared with control (NT: non-treatment control, Figure 1B). Next, the inhibitory effects of those 10 compounds on PANC-1 cell viability after a 48-h incubation were evaluated at concentrations of 0.3, 3, 10, and 30 μM (Figure 1C). 

**Figure 2:**
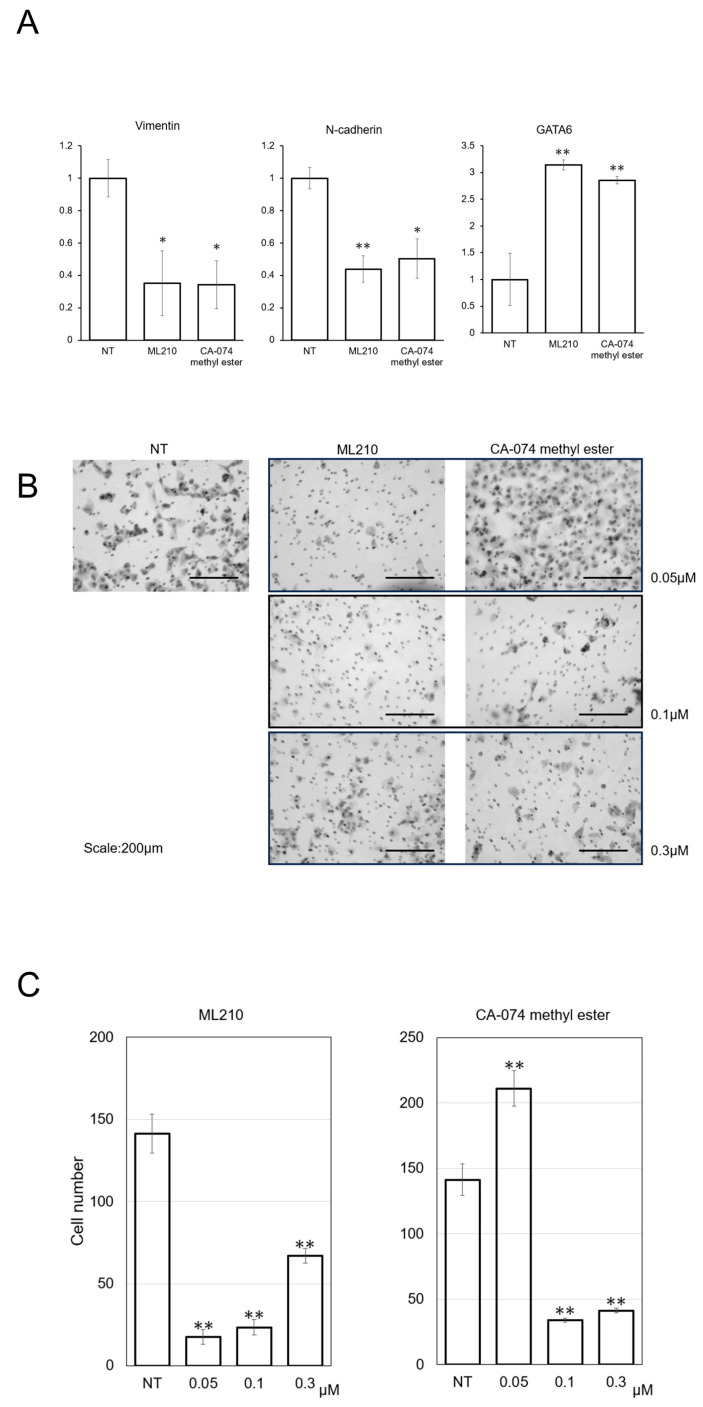
(**A**) Vimentin, N-cadherin, and GATA6 gene expression analyzed by real-time qPCR. A total of 10,000 cells were seeded in a 96-well dish with 100 µL of culture medium, and we replaced the medium with each chemical compound after 24 h. Each drug was used at the concentration of 3 µM, and the evaluation was performed 48 h later. (**B**,**C**) Diff-Quik staining images and the graphs of quantified cells that were treated with 0.05/0.1/0.3 μM ML210 or CA-074 methyl ester the after migration assay was performed. Migrated cells were counted in × 200 images (*n* = 5). Scale bar: 200 μm. Mean ± SD data are displayed as fold changes relative to the non-treated group. * *p* < 0.05, ** *p* < 0.01 (vs. the non-treated group).

Ultimately, we focused on 2 compounds, ML210 (CID 49766530) and CA-074 methyl ester, since they both showed strong inhibitory effects on cell viability and expression of vimentin and N-cadherin (Figure 2A). Low GATA6 expression, which is known as a basal-like type characteristic was found in the NT group and both treatments increased the expression of GATA6 (Figure 2A). To evaluate their inhibitory effects on EMT, we performed a migration assay (Figure 2B). Although ML210 and CA-074 methyl ester both inhibited cell migration, ML210 exerted stronger inhibitory effects at 0.05 μM (Figure 2B,C).

### 3.2. ML210 Exerts Anti-Tumor Effects in Combination with GEM

**Figure 3:**
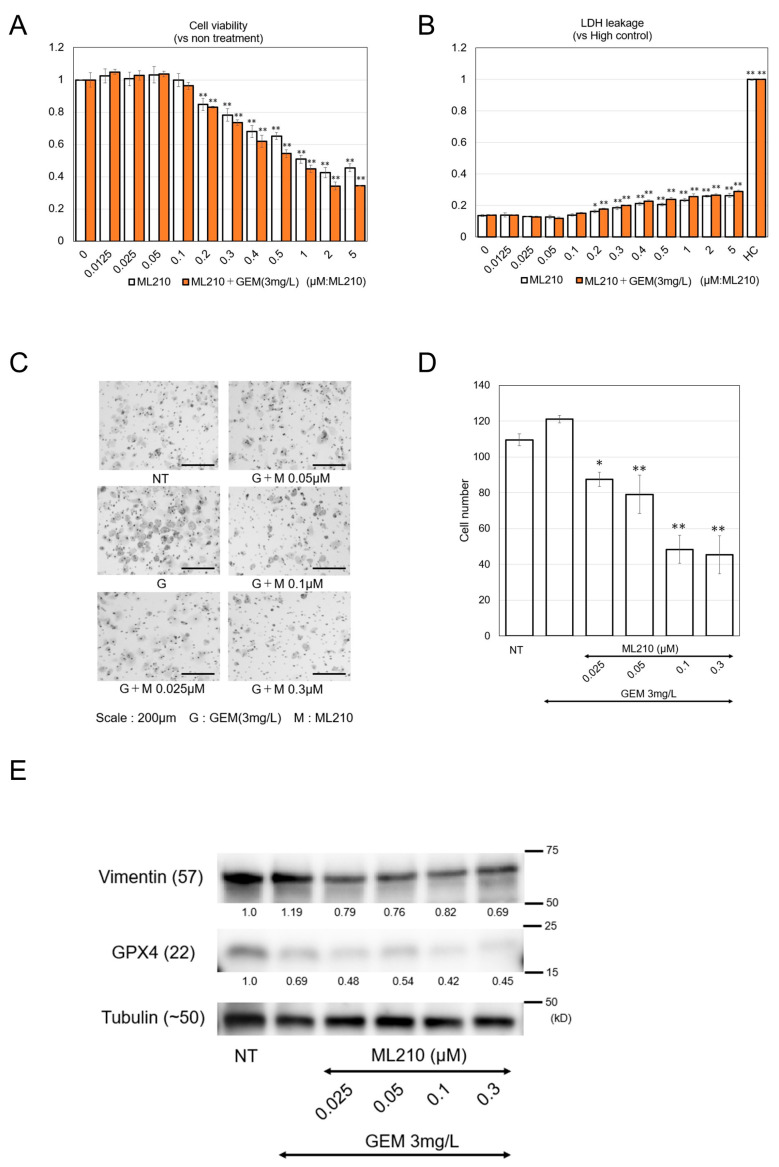
(**A**) Cell viability of PANC-1 cells (vs the non-treatment control). A total of 5000 cells/well (100 μL) were treated with 3mg/L GEM or 3 mg/L GEM + ML210 at the indicated doses. Data were obtained from three independent experiments. (**B**) LDH leakage of PANC-1 cells (vs the high control (HL)). A total of 5000 cells/well (100 μL) were treated with 3 mg/L GEM or 3mg/L GEM + ML210 at indicated doses. (**C**,**D**) Diff-Quik staining images and the graphs of quantified cells that were treated with 3 mg/L GEM or 3 mg/L GEM + 0.025/0.05/0.1/0.3 μM ML210 after migration. Migrated cells were counted in x 200 images (*n* = 5). Scale bar: 200 μm. (**E**) The protein expression of vimentin, GPX4, and tubulin were measured by western blotting analysis. PANC-1 cells were pretreated with 3 mg/L GEM and 0.025/0.05/0.1/0.3μM ML210 for 48 h. Numbers indicated below the blotting bands are quantified protein levels relative to the non-treatment control. Western blotting original images can be found in Supplementary figure. Mean ± SD data are displayed as fold changes relative to the non-treated group. * *p* < 0.05, ** *p* < 0.01 (vs. the non-treated group).

Although both ML210 and CA-074 methyl ester suppressed EMT markers, vimentin and N-cadherin, and increased GATA6, ML210 in particular exerted the strongest inhibitory effects on cell proliferation and cell migration (Figure 1C and Figure 2A–C). In addition, a cell viability assay revealed that ML210 did not suppress cell viability up to a dose of 0.1 μM (Figure 3A), which is in contrast to the lower dose of 0.05 μM that clearly inhibited cell migration (Figure 2B,C).

The optimal concentration for NAC may not affect cell viability but may be effective against migration. We decided to focus on ML210 thereafter and found that it did not show apparent toxicity up to a dose of 0.1 μM, as assessed by a lactate dehydrogenase (LDH) leakage assay (Figure 3B). Because curative surgery is scheduled after completion of NAC, it is critical to avoid severe adverse effects and to keep the body in good condition during NAC; therefore, ML210 may be a suitable agent for NAC.

GEM is widely used for PDAC treatment, including treatment of elderly patients, since it does not have strong adverse effects [17]. However, the mild anti-tumor effect of GEM against PDAC is not sufficient; thus, further development is required for effective therapy [17]. We decided to test whether the combination of ML210 with GEM exerts potent anti-tumor effects on PDAC (Figure 3A). ML210 in combination with GEM exerted stronger anti-tumor effects on PDAC cells than ML210 alone. Importantly, the combination of ML210 with GEM did not show apparent toxicity, as assessed by LDH leakage, up to a dose of 0.1 μM (Figure 3B).

### 3.3. ML210 Suppresses EMT When Given in Combination with GEM, but Does Not Induce Cell Death in PDAC Cells at Low Concentrations

Next, we confirmed the EMT phenotype and found that the combination of GEM+ML210 profoundly suppressed cell migration in a dose-dependent manner (Figure 3C,D). Combination therapy also reduced the protein expression of vimentin, along with glutathione peroxidase 4 (GPX4) (Figure 3E).

**Figure 4:**
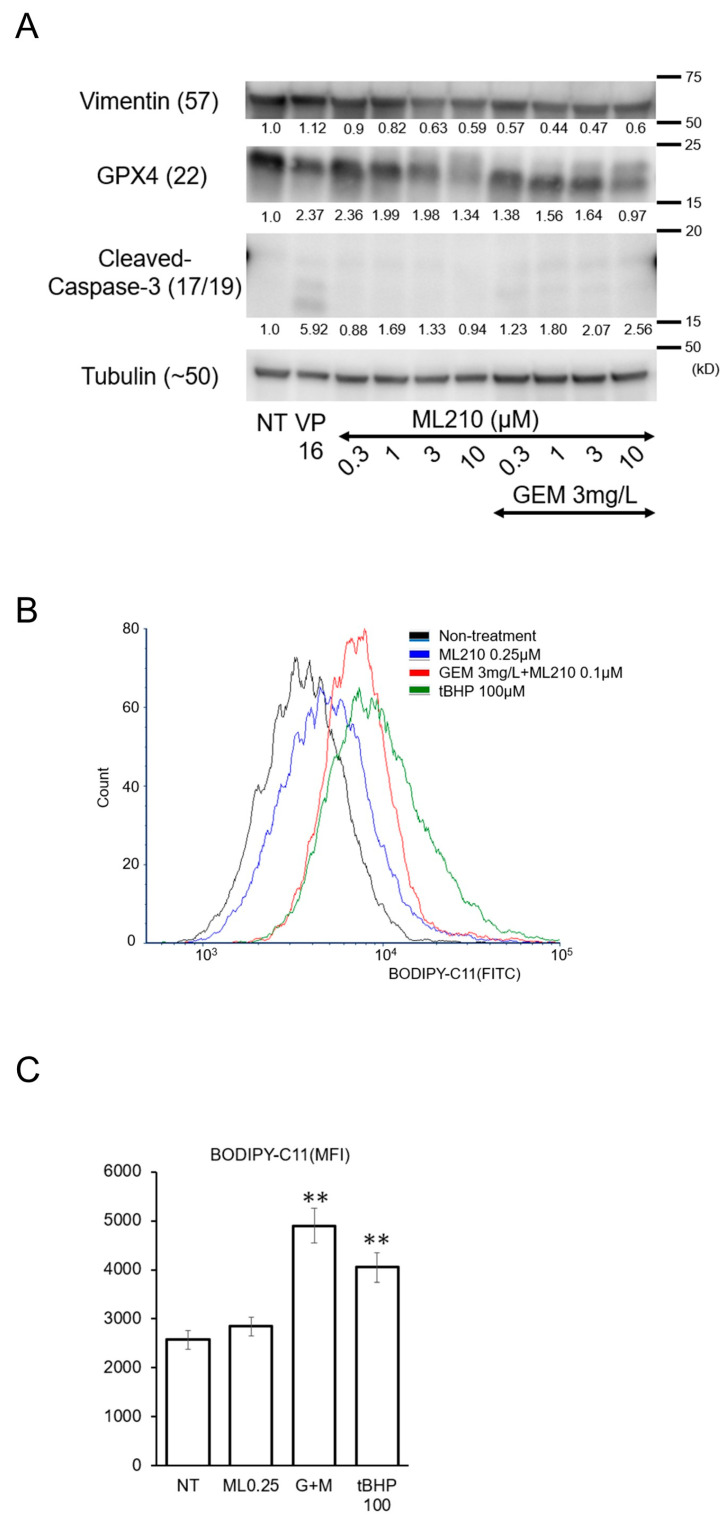
(**A**) The protein expression of vimentin, GPX4, cleaved caspase-3, and tubulin were measured by a western blotting assay. PANC-1 cells were treated with VP16 or 3mg/L GEM and/or ML210 at the indicated concentrations. VP16, or etoposide, is known as an apoptosis inducer. Numbers indicated below the blotting bands are quantified protein levels relative to the non-treatment control. Western blotting original images can be found in Supplementary figures. (**B**,**C**) Flow cytometer analysis of BODIPY581/591 C11 positive cells to detect lipid peroxidation and the graphs of data obtained from five independent experiments. The cells were treated with 0.25 μM ML210, 3 mg/L GEM + 0.1 μM ML210, or 100 μM Tert-butyl hydroperoxide (tBHP), which is an oxidative stress inducer that was used as a positive control drug. After a 24 h incubation with the indicated drugs, the positive cells were evaluated. Mean ± SD data are displayed. ** *p* < 0.01 (vs. the non-treated group).

At 0.2 μM, ML210 showed inhibitory effects on cell proliferation, cell migration, and vimentin expression in combination with GEM. Since ML210 was developed as a ferroptosis inducer owing to its suppression of GPX4 [18], we examined whether ML210 induces cell death at higher doses. Interestingly, ML210 alone or in combination with GEM, did not cause apparent cell death based on the assessment of cleaved caspase-3, even up to a dose of 10 μM (Figure 4A). Notably, VP16, also known as etoposide, induced cell death in PANC-1 cells. ML210 at low concentrations around 0.05 μM clearly suppressed cell migration (Figure 2C), but did not suppress cell viability (Figure 3A) or induce apparent cell toxicity (Figure 3B) up to 0.1 μM. Microscopic observation showed that cell proliferation was arrested weakly at 0.5 μM or more, but there were no obvious signs of cell death, such as necrotic bodies, apoptotic bodies, or cell shrinkage at that concentration.

### 3.4. Mechanism of ML210-Induced EMT Suppression

Since GPX4-specific inhibition by ML210 increases lipid peroxides in cell membranes [19], a further increase when given in combination with GEM for the treatment of PDAC was expected. Cancer cells with RAS mutations, which are present in almost all patients with PDAC, are known to be sensitive to ML210 [20].

Even though cell death, including ferroptosis, was not induced by ML-210 in PANC-1 cells, lipid peroxidization may have increased. To evaluate lipid peroxidation, we used BODIPY-C11 for PANC-1 cells. When PANC-1 cells were treated with ML210, BODIPY-C11-positive cells increased at 0.25 μM. The combination of ML210 and GEM further increased BODIPY-C11-positive cells at 0.1 µM, which was lower than the concentration of ML210 alone, and this effect was comparable to a positive control drug tert-butyl hydroperoxide (tBHP), which is known as an oxidative stress inducer (Figure 4B,C).

**Figure 5:**
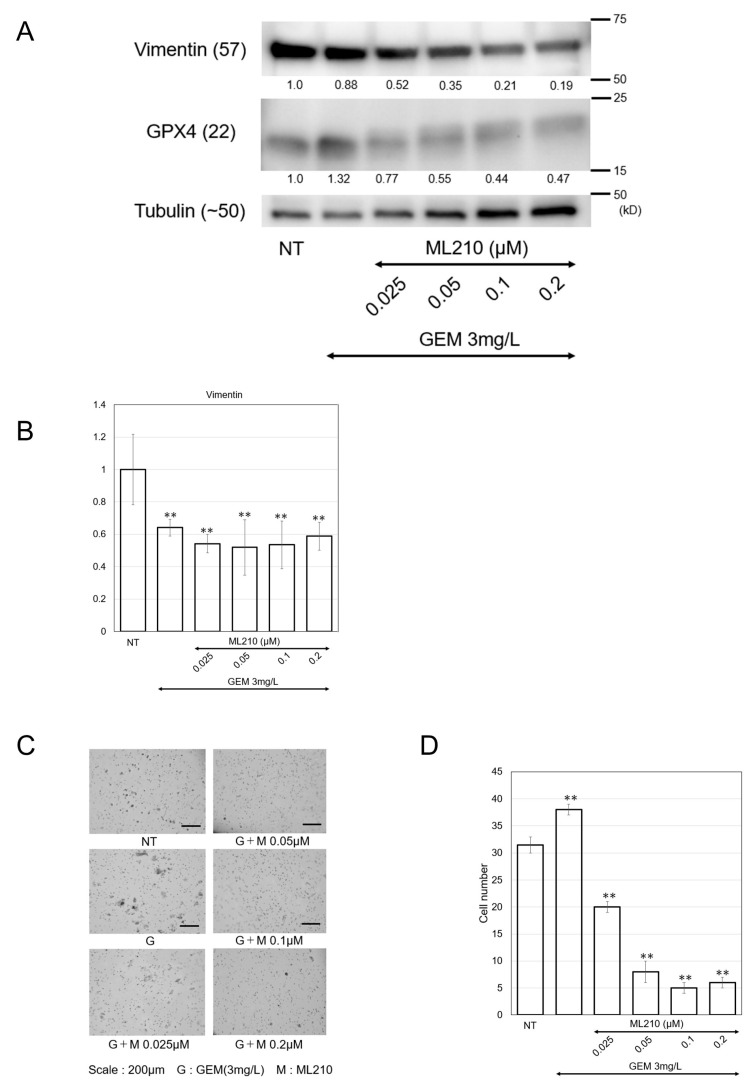
(**A**) The protein expressions of vimentin, GPX4, and tubulin were measured by western blotting analysis. MiaPaCa-2 cells were treated with 3mg/L GEM and ML210 at the indicated concentrations for 48 h. Numbers indicated below the blotting bands are quantified protein levels relative to the non-treatment control. Western blotting original images can be found in Supplementary figure. (**B**) Vimentin gene expression analyzed by real-time qPCR. (**C**,**D**) Diff-Quik staining images of the migration assay. The graphs of quantified cells that were treated with 3 mg/L GEM + 0.025/0.05/0.1/0.2 μM ML210. The invaded cells were counted in ×200 images (*n* = 5). Scale bar indicates 200 μm. Mean ± SD data are displayed as fold changes relative to the non-treated group. ** *p* < 0.01 (vs. the non-treated group).

Although the mechanism has not been clearly elucidated, MiaPaCa-2 cells, another type of PDAC cells, were analyzed. Consistent with the results of the PANC-1 cell analysis, the protein expression of vimentin was comparable with GEM treatment, then reduced with combination therapy (G + M) along with GPX4 reduction (Figure 5A). ML210 and GEM also showed strong inhibitory effects on the mRNA expression of vimentin (Figure 5B). We assessed the EMT phenotype and found that the G+M combination profoundly suppressed cell migration in a dose-dependent manner (Figure 5C,D).

## 4. Discussion

### 4.1. Treatment of PDAC Is a Difficult Challenge

Better prognosis can be achieved only if the disease is detected early enough to perform a curative pancreatectomy. In fact, many cases are treated with anti-tumor drugs because of a lack of indication for surgery. Since preoperative chemotherapy (NAC) for resectable pancreatic cancer improved postoperative recurrence and survival in the Prep-02/JSAP05 study, two courses of NAC using GEM + S-1 (GS regimen) has become the standard [9]. Although NAC has become the standard, there are cases in which surgery is not indicated due to liver metastasis or other stage progression during the waiting period. Although the GS regimen for NAC was reported to be useful, even after curative resection, postoperative recurrence often occurs due to liver metastasis, peritoneal dissemination, or lymph node metastasis [5,6].

Therefore, although GS has become the standard regimen for NAC, it is still not sufficient, and a better NAC treatment regimen is long awaited.

### 4.2. ML210, a GPX4 Inhibitor, Has Inhibitory Effects on EMT

Screening to identify a drug that inhibits EMT of pancreatic cancer cells led to the selection of ML210, a GPX4 inhibitor. ML210 inhibited EMT at low concentrations without apparent cytotoxicity. This study showed that ML210 was effective and safe in combination with GEM, suggesting that this combination may be useful as a NAC regimen.

Specifically, GPX4 may contribute to the survival of pancreatic cancer cells through the following functions [20]: 1. Inhibition of lipid peroxidation: GPX4 inhibits the induction of ferroptosis by preventing oxidative damage to plasma membrane lipids. 2. Promotion of cancer cell survival: GPX4 activity is essential for the survival of many cancer cells, including pancreatic cancer cells. 3. Treatment resistance: pancreatic cancer cells with high GPX4 expression and activity may be resistant to treatments that induce ferroptosis. Therefore, it was expected that ML210 would cause ferroptosis in PANC-1 cells.

In our study, ML210 did not induce ferroptosis; however, it inhibited EMT, including migration via increasing lipid peroxidation. Although there are no reports clearly describing the relationship between GPX4 and EMT, we found reports on the relationship between lipid peroxidation and cell migration. In retinal diseases, inhibition of lipid peroxidation suppressed EMT-driven cell migration, while the treatment did not influence the cell viability or proliferation, which is in agreement with our findings [21].

### 4.3. ML210 Specifically Inhibits GPX4 and Was Developed Primarily to Induce Ferroptosis

However, recent studies have reported the existence of factors that suppress ferroptosis independently of GPX4. Ferroptosis, a cell death process driven by iron-dependent phospholipid peroxidation, has been implicated in a variety of diseases. One surveillance mechanism for inhibiting ferroptosis is GPX4, which catalyzes the reduction of phospholipid peroxides. ML210, a GPX4 inhibitor, is expected to induce ferroptosis. In this study, low concentrations of ML210 promoted lipid peroxidation and suppressed EMT in pancreatic cancer cells; however, ML210 did not cause cell death at doses up to 10 μM (Figure 4A). In conclusion, ML210 inhibits migration without causing ferroptosis in pancreatic cancer cells.

The following are possible reasons why pancreatic cancer cells show resistance to ferroptosis due to GPX4 inhibition: high expression of FSP1, low expression of OPA1, and impaired autophagy function.

In contrast, a protein called FSP1 (ferroptosis suppressor protein 1) has been found to produce metabolites with free radical-induced antioxidant activity and has been shown to suppress ferroptosis. FSP1 may suppress ferroptosis independently of GPX4 in our study [22,23,24]. Regarding the contributions of the mitochondria, optic atrophy 1 (OPA1), a mitochondrial dynamin-like GTPase, confers susceptibility to ferroptosis by maintaining mitochondrial homeostasis and function [25]. Autophagy is reported to promote ferroptosis in the development of acute pancreatitis [26]. Lack of OPA1 or autophagy dysfunction may have prevented ferroptosis in this study.

### 4.4. Advantages of Using ML210

Because curative surgery may be performed later, it is critical to avoid adverse effects and to keep the body in good condition when patients with PDAC undergo NAC. ML210 is a selective covalent inhibitor of cellular GPX4, an enzyme that suppresses lipid oxidization of cell membranes [18]. ML210 was developed as a selective GPX4 inhibitor, and it can selectively kill cells.

GPX4 is known as an important factor for the growth of the spinal cord, and its inhibition causes embryonic death. Regarding cancers, it is reported that GPX4 is involved in the proliferation, migration, and apoptosis of glioma cells [27]. GPX4 is one of the essential regulators of ferroptosis [28] in gastric cancer [29], clear cell carcinoma (often occurring in the kidneys and ovaries) [30], and lung cancer [31]. BRCA1 deficiency induces ferroptosis vulnerability to PARP and GPX4 co-inhibition, which is useful for therapeutic strategies to overcome PARP inhibitor resistance in BRCA1-deficient cancers, including breast cancer [32]. Therefore, targeting GPX4 in human cancer has been an attractive theme [19].

Rather surprisingly, we found that ML210 inhibits EMT at low concentrations without cell death induction. Although little is known about the role of GPX4 in pancreatic cancer, FSP1 or lack of OPA1 or autophagy dysfunction may suppress ferroptosis independently of GPX4, such that ML210 caused EMT suppression.

Furthermore, gemcitabine monotherapy enhanced PDAC cell migration (Figure 3D and Figure 5D), which is in line with a previous report [33]. Hence, ML210 may be a potent drug that compensates for the weakness of GEM.

### 4.5. ML210 Is Susceptible to Cancer Cells Harboring RAS Mutations

More than 90% of pancreatic cancers have mutations in the KRAS gene, and it has been noted that cancers with mutant KRAS are susceptible to ferroptosis [34]. This may be related to increased reactive oxygen species (ROS) production and activation of fatty acid synthesis, such as via fatty acid synthetase [34], and pancreatic cancers with KRAS mutations may be expected to have increased lipid peroxidation from GPX4 inhibition. Specifically, ML210, which had improved potency in RAS-mutated cells compared with previously known RAS-selective compounds, was identified via a high-throughput screen of 303,282 compounds; therefore, it will be highly useful in identifying pathways that can potentially be used for selectively inhibiting cancer cells [35]. The results of our experiments suggest that ML210 may not cause ferroptosis but may suppress EMT at low concentrations in PDAC cells harboring KRAS mutations.

### 4.6. Mechanism by Which ML210 Suppresses Cell Migration

At doses up to 10 μM, ML210 alone did not cause cell death (Figure 4A), including ferroptosis, but it increased the concentration of oxidized lipids at a dose of 0.25 μM. In combination with GEM, ML210 did not induce cell death at doses up to 0.3 μM, but it increased the concentration of oxidized lipids at a dose of 0.1 μM (Figure 4B), suggesting that the increase in lipid peroxidation in the cell membrane due to the specific inhibition of GPX4 by ML210 may suppress cell migration. The further increase in lipid peroxidation due to the combination with GEM may have led to the combined effect.

### 4.7. Limitations

We used two drug libraries, 184 flavonoid- and 101 covalent-compounds, for the first screening. Although it is possible to find a larger number of suitable compounds from a larger compound library, we found ML210 by examining 285 compounds from a targeted compound library. Most drugs are clinically applied or easy to apply clinically because their targets are well-defined and their toxicity is within acceptable limits. Recently, we found an anti-fibrotic effect of proteasome inhibitors on the liver by using these libraries and reported that carfilzomib can be utilized for drug repositioning [36]. As a second example of the usefulness of drug libraries, this study revealed ML210 could be a strong potential candidate for improving the treatment of pancreatic cancer.

We propose that the combination of GEM and ML210 may be suitable as a NAC regimen, but that GEM as a standard chemotherapeutic agent is not effective; the use of ML210 with other agent(s) like nab-paclitaxel may be fundamental, not only for NAC, but also for conventional chemotherapy or postoperative chemotherapy, which may require ML210 for improved outcomes. These are issues for future study.

## 5. Conclusions

In summary, we have shown that ML210 suppressed cell migration in PDAC and achieved marked inhibition of EMT when combined with the chemotherapeutic drug GEM.

This is the first study to show clearly that ML210 or GPX4 inhibitors can be effective against PDAC. Specifically, the use of low concentrations of ML210 was not toxic, even in combination with GEM, suggesting that ML210 could be a promising therapeutic agent for NAC in patients with PDAC.

## Data Availability

Data supporting this study are included within the article and/or supplementary files. The other data are available upon reasonable request.

## Supplementary Materials

The following supporting information can be downloaded at: https://www.mdpi.com/article/10.3390/biom15010070/s1, Western blotting original images and supporting data.

## Abbreviations

The following abbreviations are used in this manuscript.

NAC	neoadjuvant chemotherapy
PDAC	pancreatic ductal adenocarcinoma
GEM	gemcitabine
EMT	epithelial–mesenchymal transition
GPX4	glutathione peroxidase 4
