# Supplementary figures and images for: Epithelial–Mesenchymal Transition Suppression by ML210 Enhances Gemcitabine Anti-Tumor Effects on PDAC Cells
Source: Biomolecules. 2025 Jan 6;15(1):70. doi: 10.3390/biom15010070 (PMC11763895; doi:10.3390/biom15010070)

Fig.3E

## 2 Vimentin

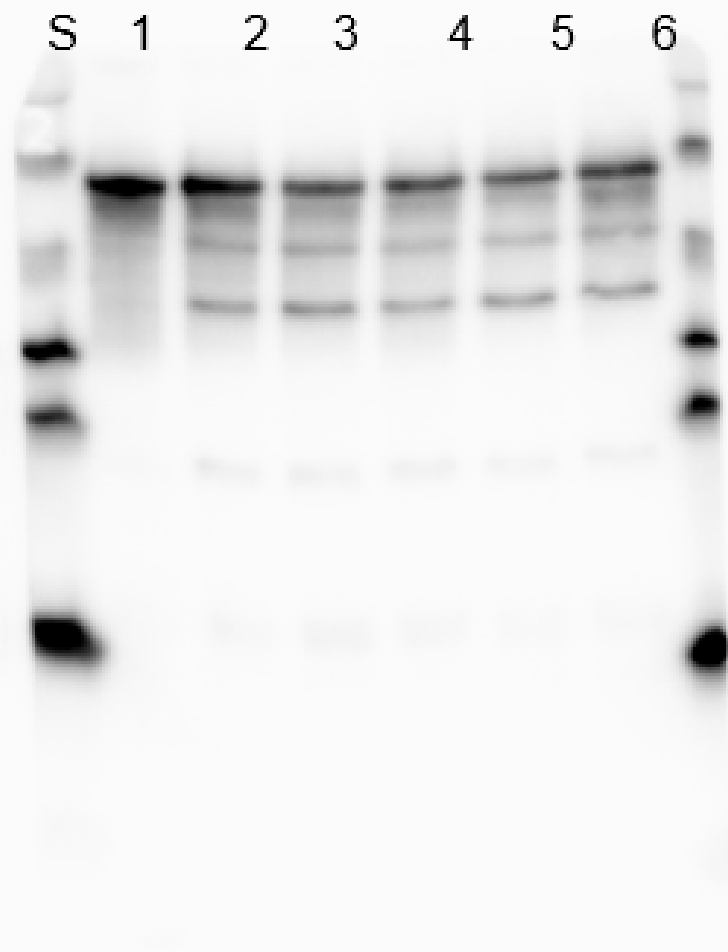

## 3 GPX4

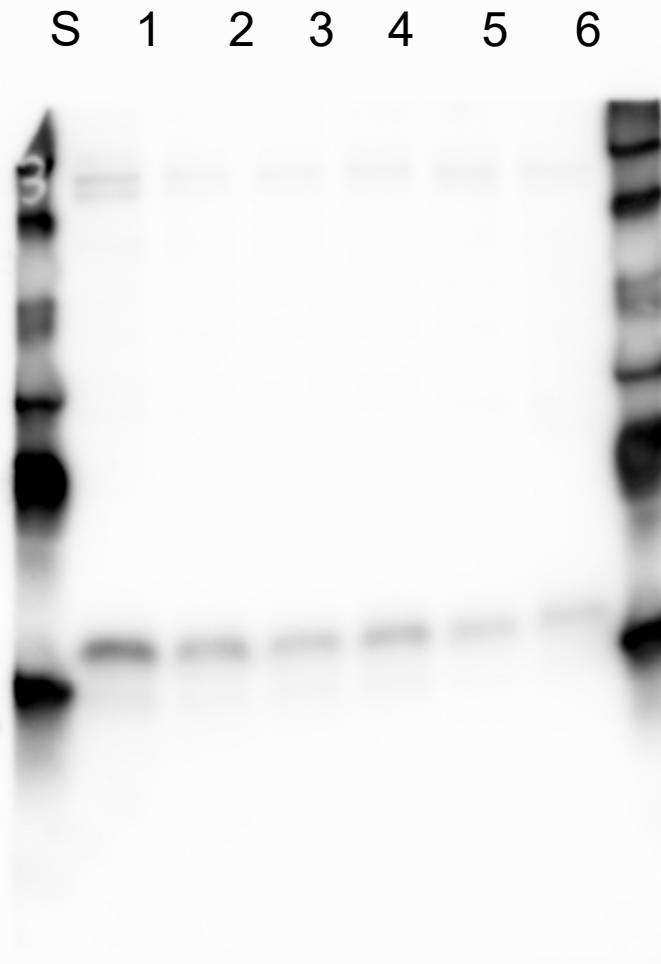

## 4 tubulin

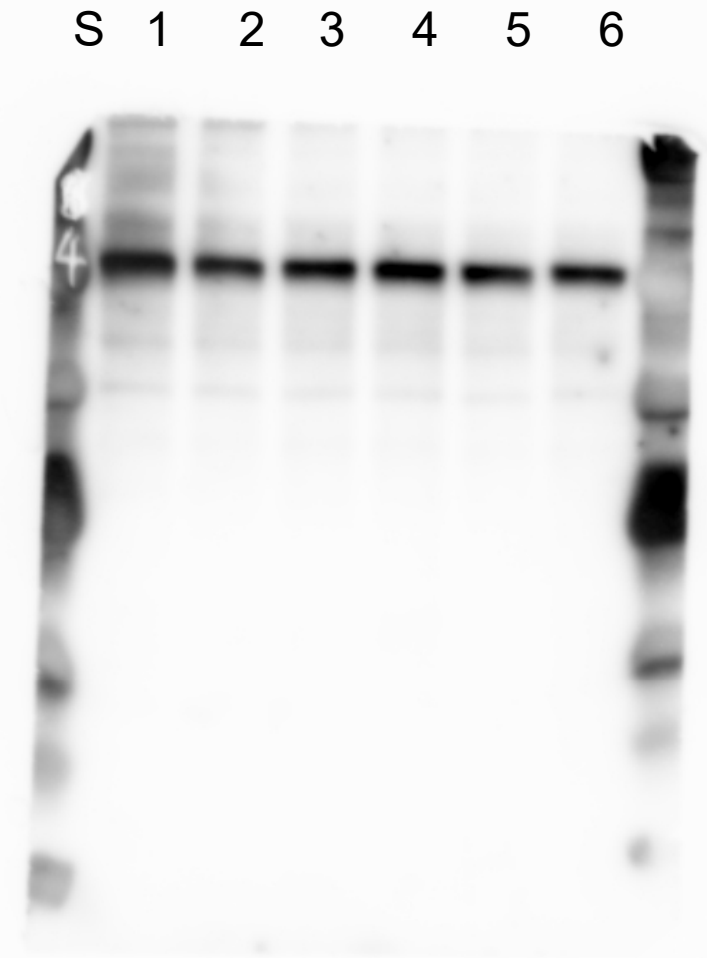

Fig.4A

Vimentin

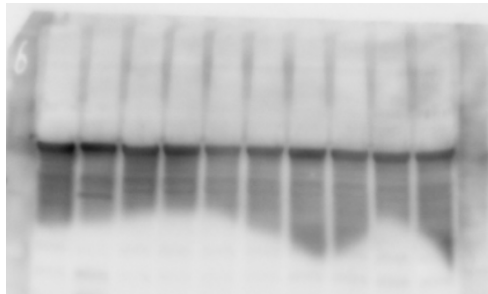

GPX4

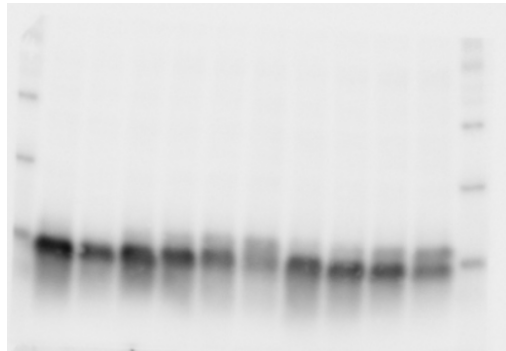

Clvd-Cas3

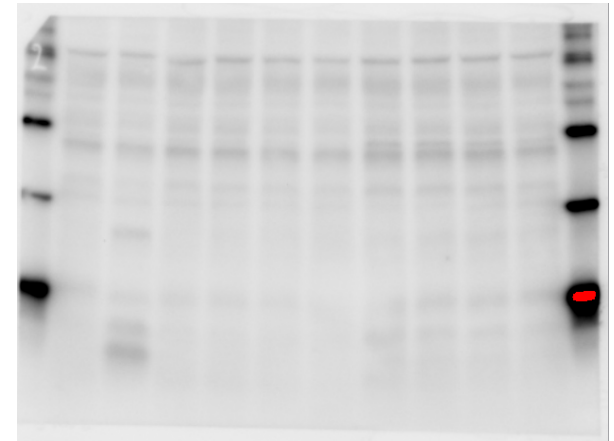

tubulin

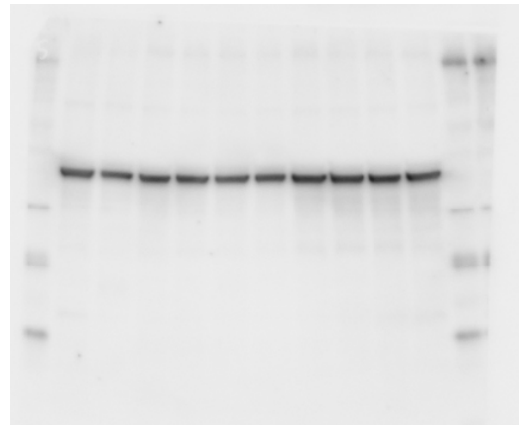

Fig.5A

Vimentin

GPX4

tubulin

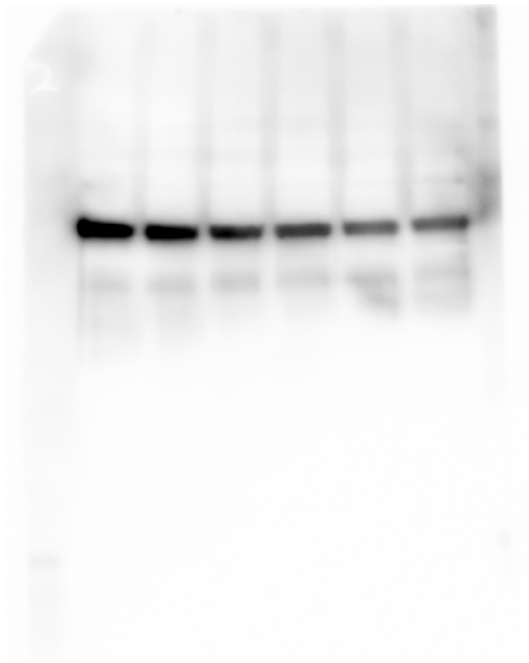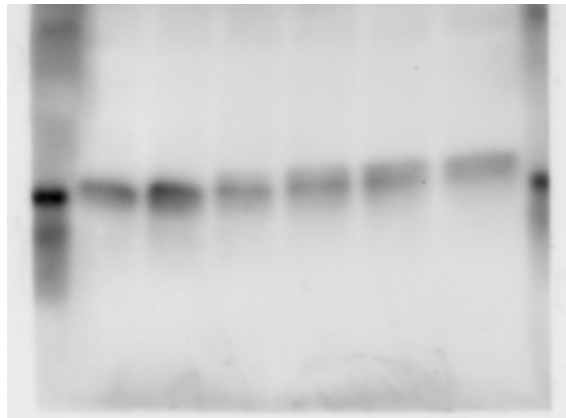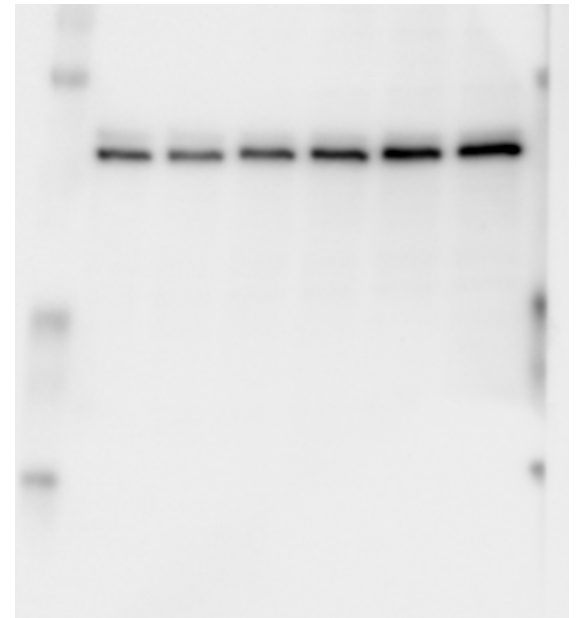

Supplement: Supplementary file 1 [file biomolecules-15-00070-s001.zip › Supplementary files/Supplementary figure1_blotting.pdf]
